# Crucial role for sensory nerves and Na/H exchanger inhibition in dapagliflozin- and empagliflozin-induced arterial relaxation

**DOI:** 10.1093/cvr/cvae156

**Published:** 2024-07-26

**Authors:** Elizabeth A Forrester, Miguel Benítez-Angeles, Kaitlyn E Redford, Tamara Rosenbaum, Geoffrey W Abbott, Vincenzo Barrese, Kim Dora, Anthony P Albert, Johs Dannesboe, Isabelle Salles-Crawley, Thomas A Jepps, Iain A Greenwood

**Affiliations:** Vascular Biology Section, Molecular & Clinical Sciences Research Institute, St George’s University, Cranmer Terrace, London SW17 ORE, UK; I Instituto de Fisiología Celular, Universidad Nacional Autónoma de México, Mexico City, Mexico; Bioelectricity Lab, Department of Physiology & Biophysics, School of Medicine, University of California, Irvine, USA; I Instituto de Fisiología Celular, Universidad Nacional Autónoma de México, Mexico City, Mexico; Bioelectricity Lab, Department of Physiology & Biophysics, School of Medicine, University of California, Irvine, USA; Department of Neuroscience, Reproductive Sciences and Dentistry, University of Naples Federico II, Naples, Italy; Department of Pharmacology, Oxford University, Oxford, UK; Vascular Biology Section, Molecular & Clinical Sciences Research Institute, St George’s University, Cranmer Terrace, London SW17 ORE, UK; Biomedical Sciences, Panum Institute, University of Copenhagen, Copenhagen, Denmark; Vascular Biology Section, Molecular & Clinical Sciences Research Institute, St George’s University, Cranmer Terrace, London SW17 ORE, UK; Biomedical Sciences, Panum Institute, University of Copenhagen, Copenhagen, Denmark; Vascular Biology Section, Molecular & Clinical Sciences Research Institute, St George’s University, Cranmer Terrace, London SW17 ORE, UK

**Keywords:** Sodium/glucose transporter 2, Sodium/hydrogen exchanger, Calcitonin-gene related peptide, Sensory nerves, Vasodilatation

## Abstract

**Aims:**

Sodium/glucose transporter 2 (SGLT2 or SLC5A2) inhibitors lower blood glucose and are also approved treatments for heart failure independent of raised glucose. Various studies have showed that SGLT2 inhibitors relax arteries, but the underlying mechanisms are poorly understood and responses variable across arterial beds. We speculated that SGLT2 inhibitor-mediated arterial relaxation is dependent upon calcitonin gene-related peptide (CGRP) released from sensory nerves independent of glucose transport.

**Methods and results:**

The functional effects of SGLT1 and 2 inhibitors (mizagliflozin, dapagliflozin, and empagliflozin) and the sodium/hydrogen exchanger 1 (NHE1) blocker cariporide were determined on pre-contracted resistance arteries (mesenteric and cardiac septal arteries) as well as main renal conduit arteries from male Wistar rats using wire myography. SGLT2, CGRP, TRPV1, and NHE1 expression was determined by western blot and immunohistochemistry. Kv7.4/5/KCNE4 and TRPV1 currents were measured in the presence and absence of dapagliflozin and empagliflozin. All SGLT inhibitors (1–100 µM) and cariporide (30 µM) relaxed mesenteric arteries but had negligible effect on renal or septal arteries. Immunohistochemistry with TRPV1 and CGRP antibodies revealed a dense innervation of sensory nerves in mesenteric arteries that were absent in renal and septal arteries. Consistent with a greater sensory nerve component, the TRPV1 agonist capsaicin relaxed mesenteric arteries more effectively than renal or septal arteries. In mesenteric arteries, relaxations to dapagliflozin, empagliflozin, and cariporide were attenuated by the CGRP receptor antagonist BIBN-4096, depletion of sensory nerves with capsaicin, and blockade of TRPV1 or Kv7 channels. Neither dapagliflozin nor empagliflozin activated heterologously expressed TRPV1 channels or Kv7 channels directly. Sensory nerves also expressed NHE1 but not SGLT2 and cariporide pre-application as well as knockdown of NHE1 by translation stop morpholinos prevented the relaxant response to SGLT2 inhibitors.

**Conclusion:**

SGLT2 inhibitors relax mesenteric arteries by promoting the release of CGRP from sensory nerves in a NHE1-dependent manner.

## Graphical Abstract

**Graphical Abstract:**
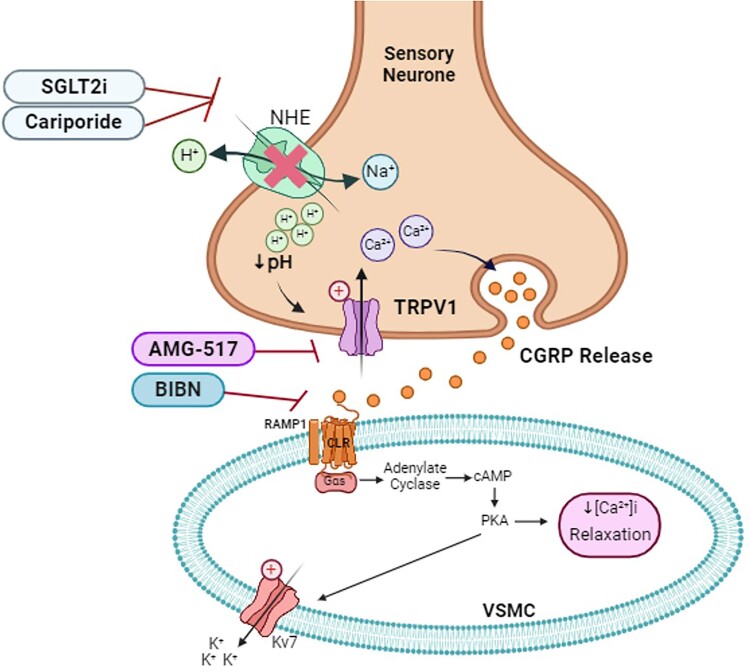
Pathway of SGLT2 inhibitor-induced relaxation in mesenteric arteries. SGLT2 inhibitors act on the sodium/hydrogen exchanger (NHE) to induce vasorelaxation. H, hydrogen; Na, sodium; Ca^2+^, calcium; CGRP, calcitonin gene-related peptide; TRPV1, transient receptor potential vanilloid 1; CLR, calcitonin receptor-like receptor; RAMP1, receptor activity-modifying protein 1; cGMP, cyclic guanosine monophosphate; PKA, protein kinase A; Kv7, voltage-gated potassium channel.

**Time of primary review: 37 days**

## 1. Introduction

Inhibitors of sodium-dependent glucose transporter 2 (SGLT2 encoded by *SLC5A2*), such as dapagliflozin or empagliflozin,^1,2^ lower blood glucose levels through increased urinary excretion of glucose.^3^ The UK National Institute for Health and Care Excellence also recommend SGLT2 inhibitors for treatment of heart failure with reduced ejection fraction and chronic kidney disease independent of raised blood glucose.^3–5^ In addition to decreased cardiac fluid retention, reduced reactive oxygen species generation, and lessened fibrosis (summarized in Preda *et al.*^3^), the cardioprotective effect of SGLT2 inhibitors has been linked to a reduction in blood pressure (∼4 mmHg). Still, the mechanism underlying this effect is not known.^6^ Some of the initial effects may be due to a decrease in circulatory volume due to increased natriuresis, but SGLT2 inhibitors still lower blood pressure at low glomerular filtration rates.^6^ The main non-natriuretic mechanism is the relaxation of arterial smooth muscle and the associated reduction in peripheral resistance. Previous *ex vivo* studies showed that empagliflozin and dapagliflozin relaxed rabbit aortic rings, rat mesenteric arteries, and rat left anterior descending coronary artery, but the underlying mechanisms were ill defined and often contradictory. In rabbit aortic rings, dapagliflozin- and empagliflozin-mediated relaxations were impaired by protein kinase G inhibitors and the non-specific Kv channel blocker 4-aminopyridine (4-AP),^7,8^ but specific blockers of Kv subfamilies (Kv1.5, Kv2.1, and Kv7s) did not impair relaxations. In contrast, SGLT2 inhibitors relaxed mesenteric resistance arteries in an endothelium-independent manner that was sensitive to blockers of Kv1.5 and Kv7 potassium channels^9–11^ without an effect on protein kinase G. In the left anterior descending coronary arteries, a significant relaxation was only observed with a high concentration of dapagliflozin (500 µM) and the relaxation was not affected by a range of potassium channel blockers.^12^ In this study, we aimed to investigate whether SGLT2 inhibitors were acting via a shared upstream mechanism that could explain the vascular-dependent effects observed in many of the trials, as well as the artery-specific differences that have been reported.

Resistance arteries that dictate blood pressure are richly innervated with peptidergic sensory nerves.^13,14^ Release of calcitonin gene-related peptide (CGRP) from sensory nerves has a pronounced vasodilatory effect in many arteries.^15–17^ CGRP has been shown to have beneficial effects in hypertensive and heart failure patients. Animal studies suggest that CGRP has vascular-dependent and vascular-independent processes by which it could protect the vasculature and myocardium against cardiovascular dysfunction, particularly heart failure,^18^ to a similar extent as the SGLT2 inhibitors. The artery-specific effects of SGLT2 inhibitors reported previously are somewhat in line with CGRP-induced relaxation. Kv7 channels have been shown to be downstream mediators of CGRP signaling,^19–21^ and CGRP can increase NO production. The present study aimed to ascertain whether SGLT2 inhibitors promoted CGRP release from perivascular sensory nerves, which would explain not only the vascular-dependent cardiovascular protective effects of SGLT2 inhibitors but also the vascular-independent protective effects.

## 2. Methods

### 2.1 Animals

Experiments were performed on second-order mesenteric and cardiac septal resistance arteries as well as conduit renal arteries from male rats aged 11–14 weeks and weighing 175–300 g. Animals were maintained under an institutional site licence and sacrificed by a Schedule 1 method (cervical dislocation) in accordance with the UK Animal (Scientific Procedures) Act 1986; therefore, no approval from a local or university ethics review board was required. This investigation conforms to Directive 2010/63/EU of the European Parliament on the protection of animals used for scientific purposes.

### 2.2 Myography

Arteries were cut into ∼2 mm segments and mounted on 40 µm stainless steel wires in a myograph (DMT, Aarhus, Denmark). The myograph chambers contained physiological salt solution (composition in Supplementary material online, *Section S1.9*) that was bubbled with 95% oxygen and 5% carbon dioxide at 37°C. Tension in each segment was recorded using LabChart Pro Software (ADInstruments, Oxford, UK). All vessels were subject to a normalization procedure^22^ to standardize the experimental conditions, and arteries were set to an internal circumference 90% of the diameter at *in vivo* transmural pressure (13.3 or 10.3 kPa for septal arteries). Endothelial integrity was estimated by the response to 10 µM carbachol applied to arteries constricted with 10 µM of the α1-adrenoreceptor agonist, methoxamine. The endothelium was denuded by mechanical abrasion with an eyebrow hair, and effectiveness of removal was ascertained by a carbachol challenge in all experiments with septal arteries.

Arterial segments were pre-contracted with 10 µM methoxamine and dapagliflozin, empagliflozin, or mizagliflozin applied cumulatively (1–100 µM). The Ki for these agents to inhibit SGLT2 is 1.2, 3.1, and 8170 nM, respectively.^1,23^ Similar experiments were performed with the NHE1 inhibitor cariporide (1 and 30 µM), CGRP (10 pM–10 nM), and capsaicin (10 µM). To identify possible underlying mechanisms, arteries were pre-incubated with a variety of agents for 15 min including the following: solvent control dimethyl sulfoxide (DMSO), linopirdine (pan-Kv7 channel blocker, 10 µM), BIBN-4096 (CGRP receptor blocker, 1 µM), capsaicin (TRPV1 agonist, 10 µM), AMG-517 (TRPV1 blocker, 1 µM), AM0902 (TRPA1 channel blocker, 10 µM), HMR-1556 (Kv7.1 channel blocker, 10 µM), iberiotoxin (BK_Ca_ channel blocker, 100 nM), 4-AP (1 mM), tetraethylammonium (TEA, 1 mM), and glibenclamide (K_ATP_ channel blocker, 1 and 3 µM). To confirm that sensory nerve-derived mediators were involved with SGLT inhibitor responses, we used repeated challenges of the TRPV1 agonist capsaicin (10 µM) to deplete CGRP content in sensory nerves.^14,16^ Capsaicin (10 µM) was applied to relaxed arteries for 5 min followed by washout to remove any released CGRP. Two further challenges of capsaicin were applied followed by extensive washout over 10 min before contraction with methoxamine (depletion protocol).

### 2.3 Immunohistochemistry

After completion of functional myography experiments, arterial segments were fixed *in situ* in myograph chambers with 4% paraformaldehyde (J61899, Thermo Scientific) for 1 h at room temperature. Arteries were then incubated for 90 min at room temperature with blocking buffer containing permeabilization agents [1% bovine serum albumin (BSA), 0.5% Triton X-100, 0.05% Tween 20 in phosphate buffered saline (PBS), composition in Supplementary material online, *Section S1.9*] and incubated overnight at 4°C with guinea pig anti-TRPV1 (Ab10295, 1:1000, Abcam), goat anti-CGRP (Ab36001, 1:1000, Abcam), rabbit anti-NHE1 (PA5115917, 1:1000, Invitrogen), mouse anti-SGLT2 (sc-393350, 1:200, Santa Cruz), rabbit anti-smooth muscle myosin heavy chain 11 (ab125884, 1:500, Abcam), and goat anti-mouse CD31/PECAM-1 (AF3628-SP, 1:150, R&D Systems) diluted in blocking buffer. This was followed by a secondary antibody incubation with either goat anti-guinea pig (Alexa Fluor 488, A11073, Life Technologies), donkey anti-goat (Alexa Fluor 633, A21082, Life Technologies), donkey anti-rabbit (Alexa Fluor 488, A21206, Life Technologies), or donkey anti-mouse (Alexa Fluor 594, A21203, Invitrogen) diluted in blocking buffer for 90 min at room temperature. Arteries were then placed in mounting medium (Vectashield Plus Antifade, Vector Laboratories) and laid flat between two glass coverslips. Arteries were excited at 405, 488, 536, and 635 nm, and fluorescence was acquired through a water immersion objective (1024 × 1024 pixels; ×40, 1.15 NA objective, Olympus) using a FV1000 laser scanning confocal microscope (Olympus, Southend-on-Sea, UK). Z-stacks were acquired through each artery wall in 1 µm increments using Fluoview (version 4.1, Olympus) software and analysed offline using Imaris (version 8.0.2, Bitplane) software.

### 2.4 Western blot and immunocytochemistry

SGLT2 expression was identified by western blot and immunocytochemistry (ICC). Protein lysates were prepared from whole mesenteric arcade, right and left renal arteries, and a combination of septal and left anterior descending artery using Triton buffer (Fisher Scientific) supplemented with protease and phosphatase inhibitors (cOmplete, mini, and PhosSTOP from Roche). Protein concentrations were determined via the Pierce™ BCA Protein Assay Kit (Thermo Fisher Scientific, Loughborough, UK). Ten micrograms of each artery sample was run under reducing conditions with 4–12% Bolt™ Bis-Tris Plus pre-cast gels (Invitrogen), and proteins were transferred to a nitrocellulose membrane. Membranes were blocked for at least 0.5 h in 3% BSA–PBS and incubated overnight at 4°C with the primary SGLT2 mouse monoclonal antibody (D-6, sc-393350, 1/200 dilution, Santa Cruz). The membranes were incubated with highly adsorbed horseradish peroxidase-conjugated goat anti-mouse IgG (A16078, Fisher Scientific) for 1 h at room temperature and developed using Immobilon™ Western Chemiluminescent HRP Substrate (Millipore). Full quantification protocol is in the Supplementary material. For ICC, vascular smooth muscle cells (VSMCs) were isolated from six mesenteric branches, right and left main renal arteries, and whole septal arteries (protocol and solution composition in Supplementary material online, *Section S1.9*), and SGLT2 localization was identified using a mouse anti-SGLT2 antibody (dilution 1:200, Santa Cruz, TX, USA) with a donkey anti-mouse secondary antibody conjugated to Alexa Fluor 488 (dilution 1:100, Thermo Fisher, Paisley, UK). Wheat Germ Agglutinin (WGA) Texas Red was used as a membrane stain before permeabilization.

### 2.5 Electrophysiology

A possible direct effect of dapagliflozin and empagliflozin was assessed on currents generated by the over-expression of Kv7 and TRPV1 genes in *Xenopus laevis* oocytes and HEK293 cells, respectively. Cell culture and channel expression methods are described fully in the Supplementary material. Potassium currents generated by the expression of KCNQ4, KCNQ5, and KCNE4 (accepted molecular combination in arterial smooth muscle^24^) were recorded using two-electrode voltage clamp at room temperature using an OC-725C amplifier (Warner Instruments, Hamden, CT, USA) and pClamp10 software (Molecular Devices, Sunnyvale, CA, USA) 2–5 days after cRNA injection. TRPV1 currents were recorded from transiently transfected HEK293 cells using the patch-clamp technique in the outside-out configuration. TRPV1-dependent currents were activated using a sub-saturating concentration (250 nM) of capsaicin (see Ortíz-Rentería *et al.*^25^). Currents were low-pass filtered at 2 kHz and sampled at 10 kHz with an EPC 10 amplifier (HEKA Elektronik) and were plotted and analysed with Igor Pro (WaveMetrics Inc.). All internal and external solutions are shown in the Supplementary material online, *Section S1.9*.

### 2.6 Morpholino studies

As NHE1 knockout mice have a short lifespan,^26^ studies were performed *ex vivo* using translation-stopping morpholinos to reduce NHE1 expression with the assumption that local translation of mRNA occurred in the sensory neurites like in motor nerves.^27^ Knockdown of NHE1 in mesenteric arteries was performed by transfection with morpholino oligonucleotides targeting NHE1 or a scrambled control as described previously.^28^ All morpholino oligonucleotides (5 µM; Gene Tools, Oregon, USA) were mixed in Opti-MEM and transfected using Lipofectamine 2000 (Thermo Fisher, Paisley, UK). Arteries were then incubated in DMEM/F-12 with 1% penicillin/streptomycin for 48 h. Transfected arteries tended to lose tone in the continued presence of methoxamine so a modified protocol was used to study the effect of SGLT2 inhibitors in these conditions. Methoxamine (10 µM) was applied for 4 min followed by washout for 20 min. This was repeated, and then 30 µM empagliflozin or dapagliflozin was applied 5 min before the third application of methoxamine. Arteries were fixed *in situ* and permeabilized, and NHE1 staining was detected to ascertain NHE1 knockdown.

### 2.7 Data and statistical analysis

All values from functional experiments are expressed as mean ± standard error of the mean (SEM) with no less than five individual data points, each representing a biological repeat. Measurements of total cell florescence during ICC involved five biological repeats with a minimum of five cells to be recorded per sample. For quantification of SGLT2 protein via western blot, a minimum of three biological repeats were obtained. For functional experiments, cumulative concentration effect curves were produced, whereby the contraction produced by 10 µM methoxamine at stable tone was taken as the maximal contraction of 100%. The tone of the artery was recorded after each subsequent addition of the pharmacological agent, and the values were formulated as a percentage of the maximum contraction. Using GraphPad Prism (RRID:SCR_002798, Version 9.0.0), a transformed data set of mean values was generated using *X* = Log(*X*) to reduce representative skew. A four-parametric linear regression analysis was then performed to produce a concentration effect curve on a log(*x*) graph with the SEM. When comparing multiple groups, a two-way analysis of variance (ANOVA) was performed followed by a *post hoc* Bonferroni or Dunnett’s test. For data comparing two groups, an unpaired parametric *t*-test was performed. Significance values are represented as *P < 0.05, **P < 0.01, ***P < 0.001 and ****P < 0.0001, and data sets subject to statistical analysis contained at least five animals per group, where *N* = number of independent values.

## 3. Results

### 3.1 Expression of SGLT2 in mesenteric and renal arteries from male Wister rats

**Figure 1:**
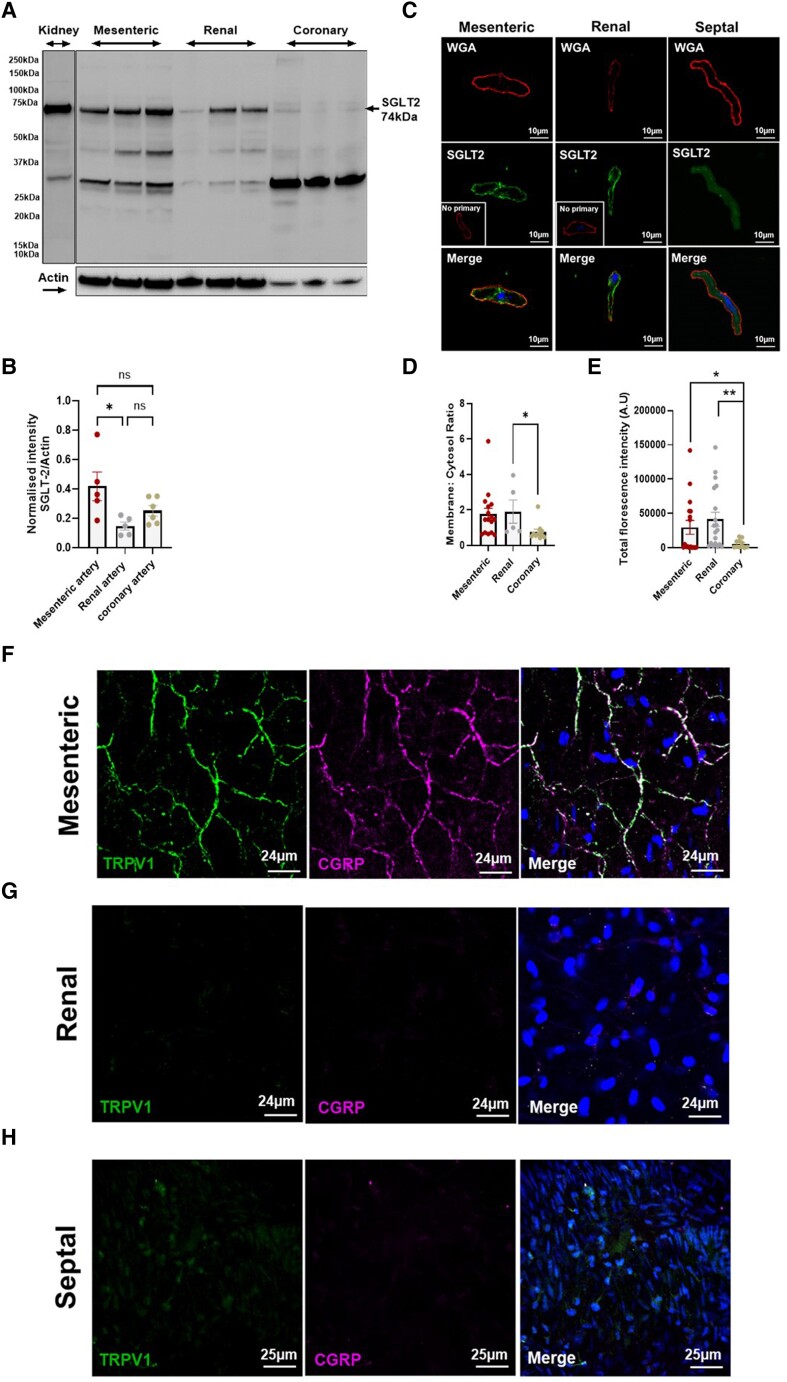
SGLT2 expression in mesenteric, renal, and coronary arteries. (*A*) Western blot quantification of SGLT2 protein in mesenteric, renal, and coronary arteries with whole kidney as a positive control (*N* = 3). Quantification of the western blot in mesenteric (left), renal (centre), and coronary (right) arteries shown in (*B*). (*C*) Representative staining of SGLT2 (middle row) and membrane stain WGA (top row) in isolated mesenteric, renal, and septal coronary VSMCs (*N* = 5, *n* = 25) with total cell fluorescence in (*D*) and membrane-to-cytosol ratio of SGLT2 expression in each artery in (*E*). All values were shown as mean ± SEM denoted by error bars, and a one-way ANOVA was used to calculate significance where *P < 0.05 and **P < 0.01. (*F*–*H*) show representative labelling for TRPV1 (green, left column) and CGRP (magenta, middle column) indicative of sensory nerve presence in the adventitia of whole mesenteric (*F*), renal arteries (*G*), and septal arteries (*H*). Nuclei were labelled in blue. Similar images seen in arteries from 4 animals). Non-significance is shown by ns.

The expression of SGLT2 within the vasculature is ill defined; hence, we characterized its expression in different vascular beds. A band at the expected molecular weight for SGLT2 (∼75 kDa) was detected in protein lysates from kidney (positive control), mesenteric, renal, and coronary arteries (*Figure 1A* and *B*). Additional bands of ∼49and ∼33 kDa were also detected corresponding to the presence of the other known isoform for SGLT2 and/or products of degradation. Immunocytochemical studies on VSMCs showed a strong co-localization of SGLT2 with the plasma membrane marker WGA, suggesting a robust expression in the membrane of VSMCs (*Figure 1C–E*). We performed immunohistochemistry experiments with well-validated CGRP and TRPV1 antibodies to delineate sensory nerves in the different arteries. *Figure 1F* shows robust TRPV1 and CGRP staining in the adventitia of mesenteric arteries with comparatively little staining in the smooth muscle or endothelial layers (see Supplementary material online, *Figure S1*). In contrast, negligible TRPV1 or CGRP staining was identified in the adventitial layer of the renal artery (*Figure 1G*; Supplementary material online, *Figure S1*), and none was detected in a septal artery (*Figure 1H*). Thus, mesenteric arteries exhibit robust sensory nerve networks that were not present in renal or cardiac septal arteries.

### 3.2 SGLT2 inhibitors relax mesenteric arteries

**Figure 2:**
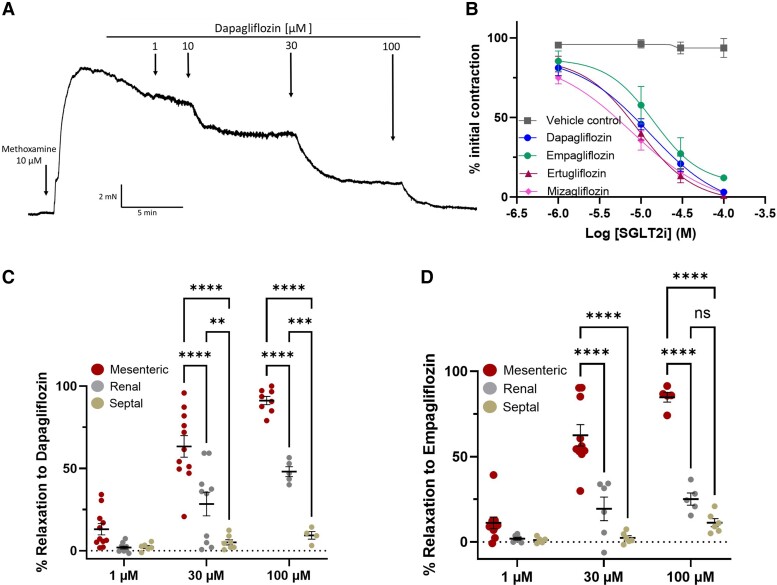
The effect of SGLT2 and SGLT1 inhibitors on mesenteric, renal, and septal arterial tones. (*A*) A representative trace of the effect of dapagliflozin in mesenteric arteries pre-contracted with 10 µM methoxamine. (*B*) Mean effect of dapagliflozin (blue), empagliflozin (green), and mizagliflozin (purple), with mean vehicle control in grey, *N*= 5–6. All values are shown as mean ± SEM denoted by the error bars (*N* = 6–8). (*C* and *D*) show the relaxation of dapagliflozin and empagliflozin in renal mesenteric (left hand data set), renal (middle data set), and septal (right hand data set) arteries. All data are individual experiments and a two-way ANOVA with a *post hoc* Sidak test was used to calculate significance values where **, ***, and **** denote *P* < 0.01, 0.001 and 0.0001 respectively. Non-significance is shown by ns.

In rat mesenteric artery segments, dapagliflozin and empagliflozin, as well as a SGLT1 inhibitor, mizagliflozin, produced concentration-dependent relaxations (1–100 µM), with approximate IC_50_ values of 9.5, 7.3 and 5.5 µM, respectively (*Figure 2A* and *B*; *n* = 5–6). The relaxation elicited by each SGLT inhibitor was not affected by the size of the induced contraction and not dependent upon a functional endothelium (see Supplementary material online, *Figures S2* and *S3*). In contrast to their effect on mesenteric arteries, dapagliflozin and empagliflozin were significantly poorer relaxants of pre-contracted renal arteries and were ineffective relaxants of septal arteries (up to 100 µM; *Figure 2C* and *D*).

### 3.3 Role of Kv7 channels in SGLT2 inhibitor-induced relaxations

**Figure 3:**
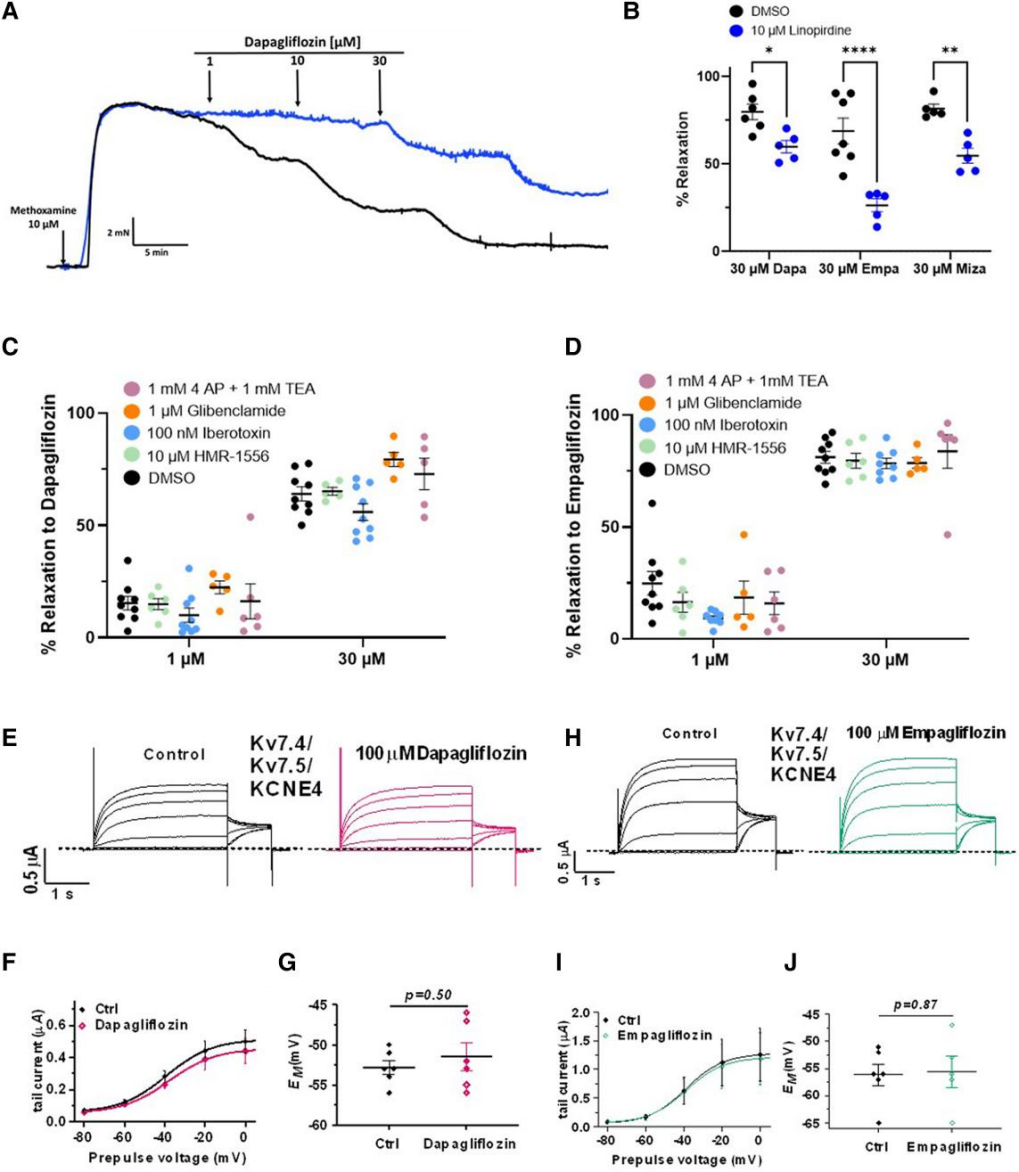
SGLT2 inhibitors and Kv7 channels. (*A*) Representative trace of the effect of dapagliflozin on pre-contracted mesenteric arteries in the presence (upper trace/blue) and absence (lower trace) of 10 µM linopirdine. (*B*) Mean data for relaxations to dapagliflozin, empagliflozin, and mizagliflozin (30 µM) in solvent control (left data set) and when pre-incubated with 10 µM linopirdine (right data set/blue) (*N* = 5–6). All values are individual experiments with mean ± SEM denoted by the error bars. A two-way statistical ANOVA with a *post hoc* Sidak test was used to generate significant values (*P < 0.05, **P < 0.01, and ****P < 0.0001). The effect of dapagliflozin (*C*) and empagliflozin (*D*) in the absence and presence of HMR1556, iberiotoxin, 4-AP, TEA, and glibenclamide (data sets left to right respectively). All data values are shown as mean ± SEM (*N* = 5–6). (*E*–*G*) show currents produced by the co-expression of Kv7.4, Kv7.5, and KCNE4 in the absence and presence of 100 µM dapagliflozin. Representative traces in (*E*), mean current–voltage relationship in (*F*), and mean membrane potential in (*G*). The effect of 100 µM empagliflozin on currents produced by co-expression of Kv7.4, 7.5, and KCNE4 is shown in (*H*–*J*). Representative traces in (*H*), mean current–voltage relationship in (*I*), and mean membrane potential in (*J*). Data are the mean of *N* oocytes with error bars denoting the SD.

Previous studies implicated Kv channels encoded by *KCNQ* genes (Kv7 channels) in the relaxant response to different SGLT2 inhibitors.^9–11^ In the present study, the relaxation of mesenteric arteries produced by dapagliflozin, empagliflozin, and mizagliflozin was significantly attenuated by pre-incubation with the pan-Kv7 channel inhibitor linopirdine (10 µM) when compared to DMSO control (*Figure 3A* and *B*; *n* = 5–6). Specific blockers of Kv7.1 (HMR1556), BK_Ca_ (iberiotoxin), K_ATP_ (glibenclamide), and the non-selective K channel blockers 4-AP and TEA had no effect on relaxations produced by SGLT2 inhibitors (*Figure 3C* and *D*; Supplementary material online, *Figure S5*). However, electrophysiological experiments on oocytes co-expressing KCNQ4/KCNQ5 and KCNE4—a molecular combination found in most arterial smooth muscle^24^ showed that neither dapagliflozin nor empagliflozin at 100 µM had any effect on the current amplitude nor the voltage dependence of activation of the Kv currents and neither agent affected the resting membrane potential (*Figure 3E–J*). Thus, dapagliflozin and empagliflozin do not activate Kv7 channels directly.

### 3.4 Sensory nerve contribution in mesenteric and renal arteries

**Figure 4:**
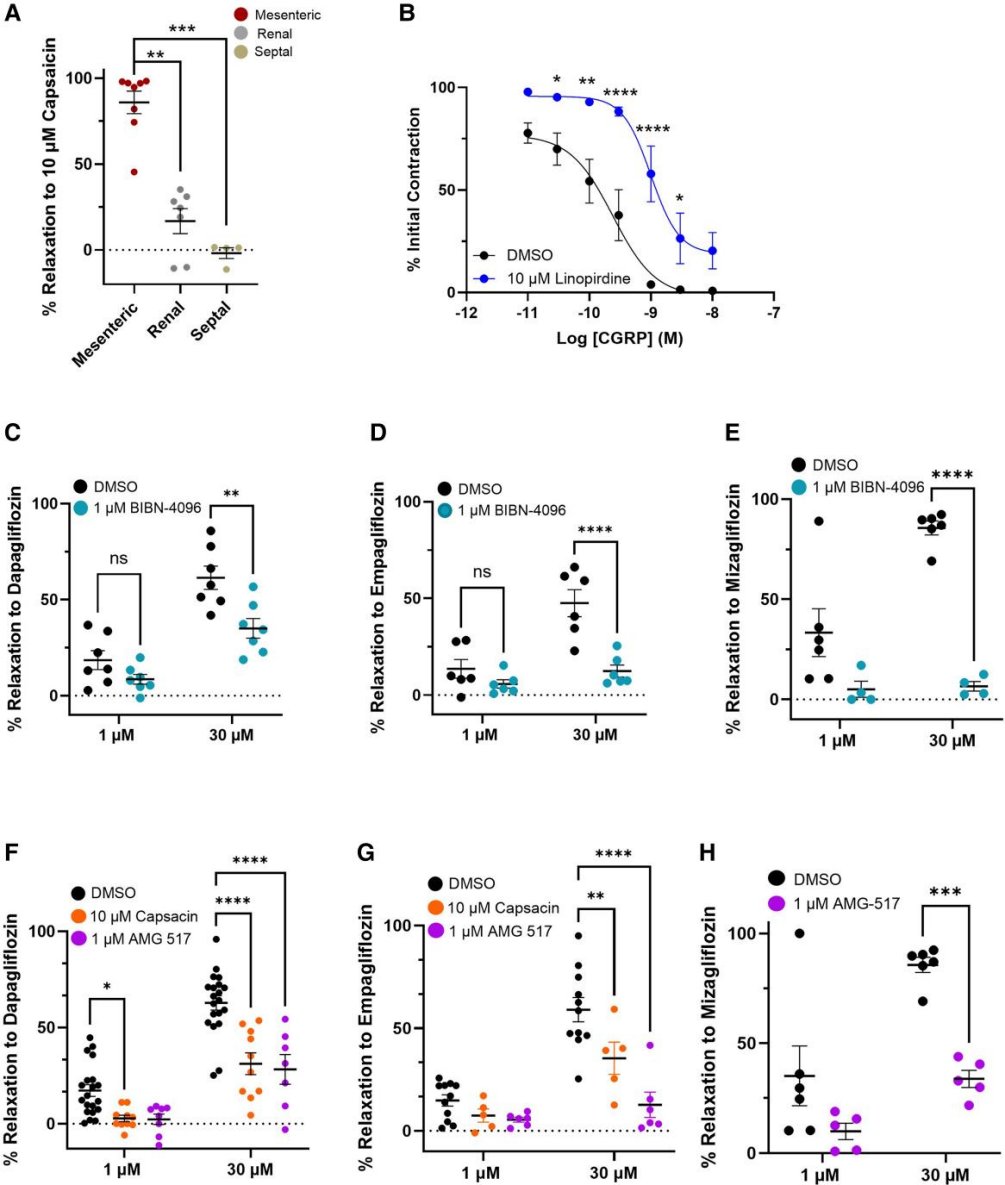
Dapagliflozin-, empagliflozin-, and mizagliflozin-induced relaxations are blocked by CGRP receptor antagonist and TRPV1 blockade. (*A*) shows the percentage relaxation to 10 µM capsaicin in mesenteric (left hand data set), renal (middle data set), and septal arteries (right hand data set) (*N* = 6–8). (*B*) shows the mean relaxation to CGRP in the absence and presence of 10 µM linopirdine (blue, *N* = 5–8). All data points are represented as mean ± SEM denoted by the error bars. A two-way statistical ANOVA with a *post hoc* Bonferroni test was used to generate significant values (*P < 0.05, **P < 0.01, ***P < 0.001, and ****P < 0.0001). The mean effect of dapagliflozin (*C*, *N* = 6), empagliflozin (*D*, *N* = 7), and mizagliflozin (*E*, *N* = 5) on pre-contracted mesenteric arteries in the presence of DMSO (left data set) and 1 µM BIBN (right data set). The mean relaxations produced by 1 and 30 µM dapagliflozin (*F*, *N* = 7–10) and empagliflozin (*G*, *N* = 6–10) in mesenteric arteries pre-incubated in DMSO (solvent control, black), 1 µM AMG-517 (right hand data set), or after sensory nerve depletion with 10 µM capsaicin (centre data set). The relaxation to mizagliflozin in theabsence and presence of TRPV1 blocker AMG-517 (right hand data set) is shown in (*H*) (*N* = 5). All values are expressed as mean ± SEM. A two-way statistical ANOVA with a *post hoc* Sidak test was used to generate significant values (*P < 0.05, **P < 0.01, ***P < 0.001, and ****P < 0.0001).

Kv7 blockers attenuate arterial relaxations produced by several agonists of Gs-linked receptors.^19–21^ As Kv7 channels are not directly activated by SGLT2 inhibitors, a logical conclusion is that these agents promoted the release of a mediator that recruited Kv7 channels. As dapagliflozin- and empagliflozin-mediated relaxations were not endothelium dependent (see Supplementary material online, *Figure S3*), we focused on CGRP released from the sensory nerves and the different effects of the SGLT2 inhibitors between mesenteric and renal or septal arcades were due to a differential abundance of sensory nerves (see *Figure 1*). CGRP can be released from sensory neurones upon TRPV1 channel activation.^14^ Applying the TRPV1 activator capsaicin fully relaxed pre-contracted mesenteric arteries (*Figure 4A*), which was abrogated by the CGRP receptor blocker BIBN-4096 (see Supplementary material online, *Figure S4A*). Consistent with the lack of sensory nerves (*Figure 1F* and *G*), capsaicin had no effect in pre-contracted renal or septal arteries (*Figure 4A*). Linopirdine inhibited mesenteric artery relaxations produced by exogenous CGRP or SGLT2 inhibitors to a similar extent (*Figure 4B*), consistent with our hypothesis that SGLT2 inhibitor-induced relaxations are mediated by CGRP.

### 3.5 Dapagliflozin- and empagliflozin-evoked relaxations are sensitive to CGRP and TRPV1 blockade

To identify a role for CGRP in SGLT2 inhibitor-induced relaxations, we applied dapagliflozin, empagliflozin, and mizagliflozin to mesenteric arteries pre-incubated with either DMSO (control) or BIBN-4096 (1 µM). The relaxation to all three agents was significantly attenuated by 1 µM BIBN-4096 (*Figure 4C–E*). To confirm that SGLT2 inhibitors relaxed mesenteric arteries through provoking CGRP release from sensory nerves, we depleted CGRP stores through treatment with three 5-min applications of capsaicin (10 µM) followed by washout of the bathing solution or directly blocked TRPV1 with AMG-517 (1 µM). Both treatments prevented the relaxation produced by 1 µM capsaicin (see Supplementary material online, *Figure S4B* and *C*). The relaxations produced by both dapagliflozin and empagliflozin were significantly attenuated after treatment with capsaicin or incubation with the TRPV1 blocker, AMG-517 (*Figure 4F* and *G*, *N* = 6–10). The mizagliflozin-induced relaxation was also sensitive to AMG-517 (*Figure 4H*). The relaxation to dapagliflozin was not affected by pre-application of the TRPA1 blocker AM0902 (1 or 10 µM, Supplementary material online, *Figure S5*). Neither the TRP blockers nor capsaicin pre-treatment affected methoxamine-induced contraction amplitude (see Supplementary material online, *Figure S6*). Hence, relaxations induced by SGLT2 inhibitors were dependent upon activation of the TRPV1 channel on perivascular sensory nerves and subsequent CGRP release.

### 3.6 Effect of SGLT2 inhibitors on heterologously expressed TRPV1 currents

**Figure 5:**
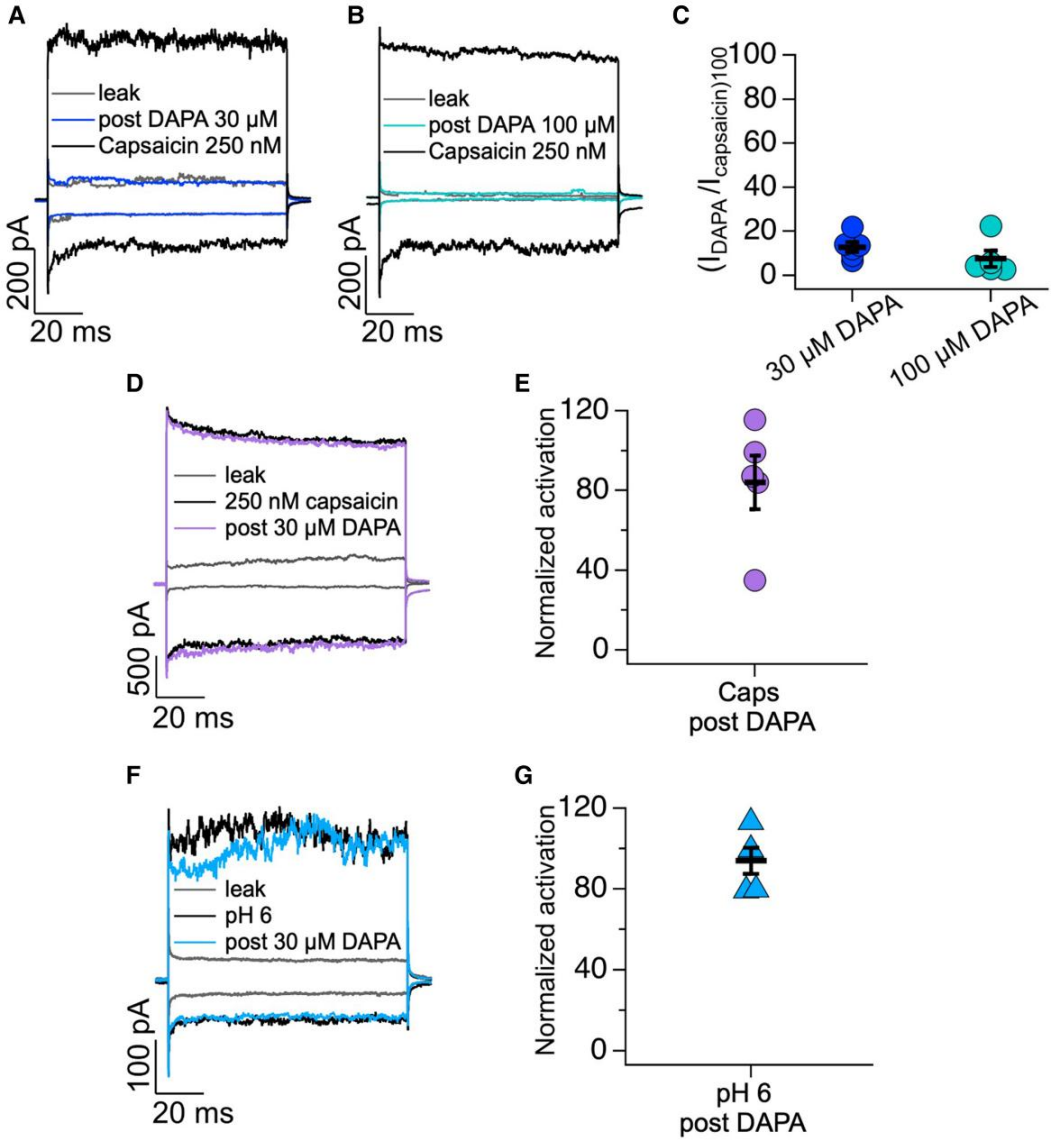
SGLT2 inhibitors do not activate TRPV1 directly. (*A* and *B*) Representative traces of currents at +120 and −120 mV from outside-out membrane patches of HEK293 cells expressing TRPV1. Leak currents were obtained in the absence of any agonist and after 5-min application of dapagliflozin (DAPA) 30 μM (blue traces, *A*) and 100 μM (green traces, *B*). The top and bottom traces shows the subsequent effect of 250 nM capsaicin. (*C*) Average data for experiments in (*A* and *B*). Currents were leak subtracted, and data were normalized to activation by capsaicin 250 nM in the steady state at +120 mV (*N* = 6 and *N* = 5 for DAPA 30 and 100 μM, respectively). (*D*) Representative traces of currents at +120 and −120 mV from outside-out membrane patches of HEK293 cells expressing TRPV1 in control conditions (grey), after application with 250 nM capsaicin (black traces) and after application of 250 nM capsaicin + 30 μM DAPA for 5 min (lilac traces). (*E*) The data in (*D*) were normalized by dividing the currents obtained at +120 mV in response to 250 nM capsaicin + 30 μM DAPA by the currents in response to 250 nM capsaicin alone; (*N* = 5). (*F*) Representative traces of currents at +120 and −120 mV from outside-out membrane patches of HEK293 cells expressing TRPV1 under control conditions (grey), after activation of TRPV1 by pH 6 (black) and after 5-min application of 30 μM DAPA to pH 6 conditions (blue). (*G*) The data in (*F*) were normalized by dividing the currents obtained at +120 mV in response to pH 6 + 30 μM DAPA by the currents in response to pH 6 alone (*N* = 6). Group data are reported as the mean ± SEM.

To determine whether dapagliflozin could activate TRPV1 channels directly, we performed patch-clamp experiments using outside-out excised membrane patches of HEK293 cells expressing rat TRPV1 (rTRPV1). As shown in *Figure 5*, we first obtained the leak currents (in the absence of agonist, grey traces) elicited by a square voltage pulse to −120 mV followed by a pulse to 120 mV, then used the same voltage protocol to assess currents after exposing the patches to either 30 μM (*Figure 5A*) or 100 μM dapagliflozin (*Figure 5B*) for 5 min and, finally, to 250 nM capsaicin alone (black traces). All currents were leak subtracted and normalized to the current obtained at +120 mV (*Figure 5C*). Currents after exposure to 30 μM dapagliflozin were 12.9 ± 2.2 and 7.6 ± 3.7% after 100 μM dapagliflozin of the currents elicited by the TRPV1 agonist, capsaicin (*Figure 5C*, *n* = 6). These data indicate that TRPV1 is not directly activated by dapagliflozin.

Next, we studied whether dapagliflozin could potentiate capsaicin- or low pH-activated TRPV1 currents. For this set of experiments, we first recorded leak currents (grey traces), then activated TRPV1 in outside-out excised membrane patches of HEK293 cells with either a sub-saturating concentration (250 nM) of capsaicin or with a solution at pH 6, then washed the membrane patches to close the channels and exposed the patches to 30 μM dapagliflozin for 5 min and remeasured the currents in the presence of 250 nM capsaicin or solution with low extracellular pH (*Figure 5D–G*). The results from these experiments indicate that 30 μM dapagliflozin did not potentiate TRPV1 currents activated by 250 nM capsaicin (*Figure 5D* and *E*; 84.1 ± 13.5%, *n* = 5) or by low pH (*Figure 5F* and *G*; 94 ± 6.5%, *n* = 5). Thus, SGLT2 inhibitors neither activate TRPV1 directly nor sensitize the channel to known mediators.

### 3.7 NHE1 co-localizes with TRPV1 and cariporide prevents SGLT2 inhibitor-induced responses

**Figure 6:**
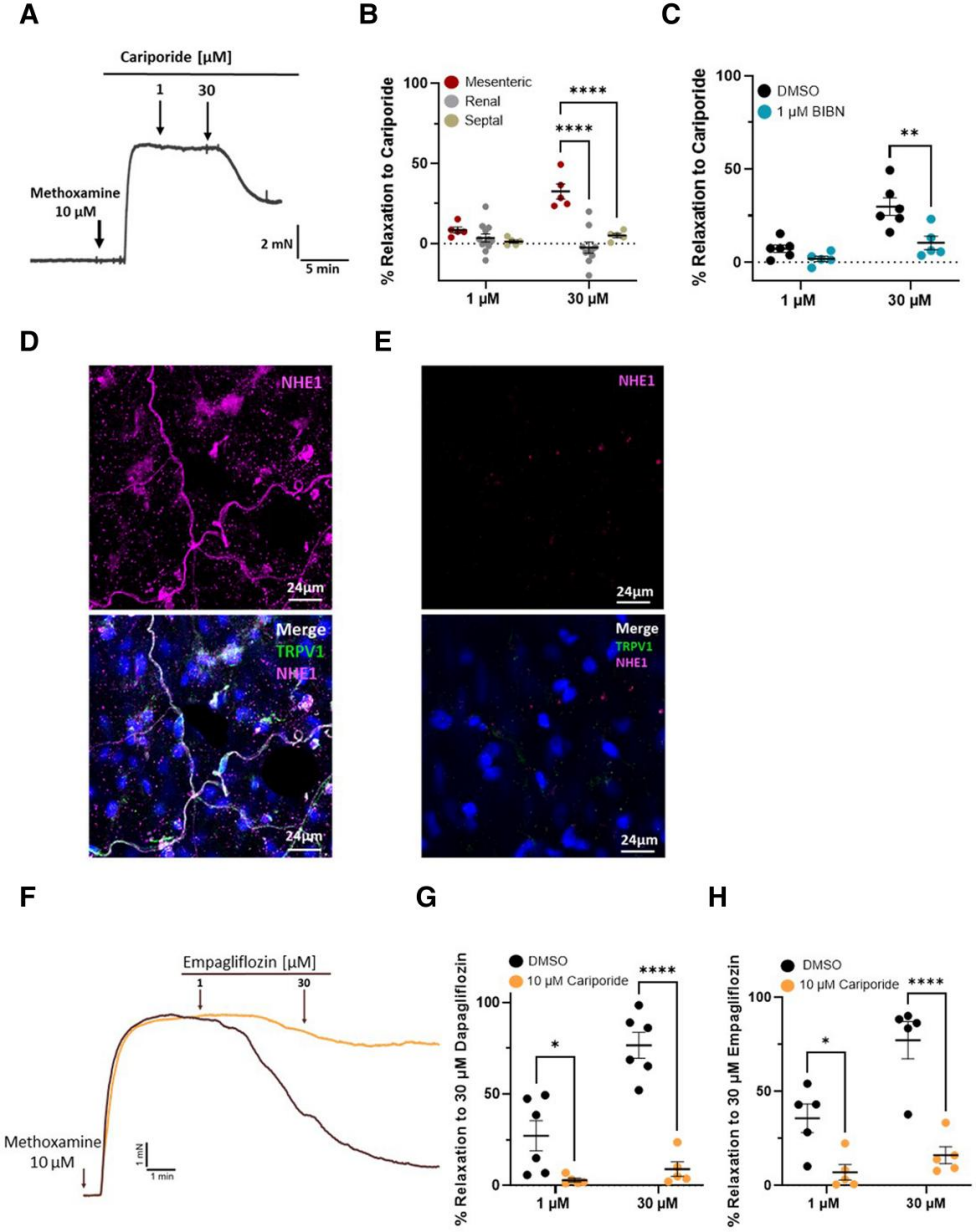
Effect of cariporide and NHE1 localization in mesenteric arteries. (*A*) A representative trace of the relaxation to 1 and 30 µM cariporide in mesenteric arteries pre-contracted with 10 µM methoxamine. (*B*) Mean percentage relaxation to 1 and 30 µM cariporide in mesenteric (left hand data), renal (middle data set), and septal arteries (right hand data set) (*N* = 6–10). (*C*) shows the mean percentage relaxation to cariporide (1–30 µM) in pre-contracted mesenteric arteries when incubated with DMSO (left) and 1 µM BIBN (right) (*N* = 5–6). (*D* and *E*) show representative labelling for TRPV1 alone (green, top) and with NHE1 (magenta, bottom) in the adventitia of whole mesenteric (*D*, *N* = 3) and renal (*E*, *N* = 4) arteries. Nuclei were labelled in blue. (*F*) Representative trace of the relaxation to empagliflozin (1–30 µM) in mesenteric arteries pre-contracted with 10 µM methoxamine in the presence (orange) and absence (black) of cariporide. The mean data for the response to 1 and 30 µM dapagliflozin (*N* = 5) and empagliflozin (*N* = 5) in the presence (top trace) and absence (bottom trace) of 10 µM cariporide are shown in (*G* and *H*). All data are individual experiments with the mean ± SEM denoted by the error bars. A two-way statistical ANOVA with a *post hoc* Sidak test was used to generate significant values (*P < 0.05, **P < 0.01, and ****P < 0.0001).

**Figure 7:**
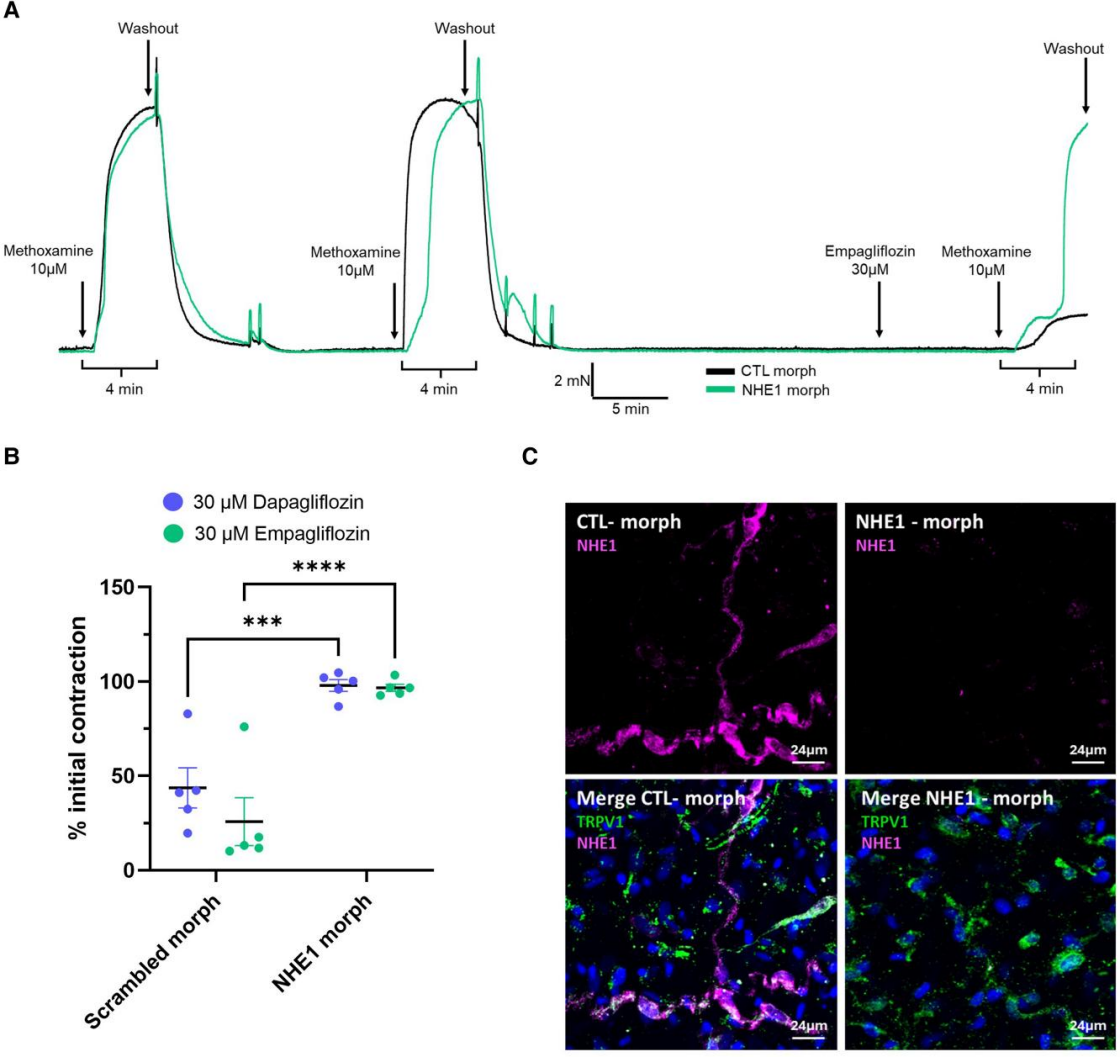
Knockdown of NHE1 impairs the relaxation of dapagliflozin and empagliflozin in mesenteric arteries. (*A*) A representative trace of the contraction to 10 µM methoxamine in the presence of 30 µM empagliflozin in scrambled control morpholino (black) and knockdown NHE1 morpholino (green/lighter line) arteries. (*B*) shows the mean data of the contraction to 10 µM methoxamine in scrambled control morpholino and knockdown NHE1 morpholino arteries when pre-incubated with 30 µM dapagliflozin or 30 µM empagliflozin. All data are represented as mean ± SEM denoted by the error bars. A two-way statistical ANOVA with a *post hoc* Sidak test was used to generate significant values (***P < 0.001 and ****P < 0.0001). (*C*) shows representative labelling for NHE1 alone (top image) and with TRPV1 (lower image) in the adventitia of whole mesenteric arteries transfected with scrambled morpholino (Ctrl) and NHE1-targeted morpholino (right hand column). Representative of 4 such experiments.

Various studies have shown that SGLT2 inhibitors block NHE isoforms^29–34^ with *in silico* studies predicting a binding site in the extracellular sodium-binding pocket of NHE1.^29^ We postulated that SGLT2 inhibitors promoted release of CGRP from sensory nerves by inhibiting NHE and producing a localized pH change sufficient to activate TRPV1. Functional experiments revealed that the NHE1 inhibitor cariporide^29^ relaxed mesenteric arteries, which were markedly impaired by BIBN-4096 or AMG-517 pre-treatment (*Figure 6A* and *C*). Cariporide was ineffective at relaxing pre-contracted renal or septal arteries (*Figure 6B*). We stained mesenteric arteries with SGLT2 and NHE1 antibodies and used TRPV1 to delineate the sensory nerves. As shown in *Figure 6D*, prominent NHE1 staining was observed in the adventitia of mesenteric arteries co-localized with TRPV1 and some staining in the smooth muscle and endothelial layers (see Supplementary material online, *Figure S7*). In contrast, negligible staining of SGLT2 was observed in the adventitial and endothelial layers, but traces of staining were identified in the smooth muscle layer of mesenteric arteries (see Supplementary material online, *Figure S7*), consistent with the staining identified in dispersed single smooth muscle cells (*Figure 1C–E*). No staining for NHE1 or SGLT2 was detected in renal artery adventitia (*Figure 6E*; Supplementary material online, *Figure S8*). Therefore, NHE1 co-localized with TRPV1 in sensory nerves in the mesenteric artery, but SGLT2 was only found in the smooth muscle layer. In mesenteric arteries, pre-treatment with 30 µM cariporide reduced the relaxations to 30 µM dapagliflozin or 30 µM empagliflozin significantly (*P* < 0.001; *Figure 6F–H*). These experiments suggest that vasodilatory effects of SGLT2 inhibitors were mediated by NHE1 inhibition. To confirm this, we used morpholino-based molecular interference to reduce NHE1 protein expression in mesenteric arteries. In control arteries transfected with non-targeting scrambled morpholinos, 5-min application of either 30 µM empagliflozin or dapagliflozin reduced contractions produced by the subsequent application of 10 µM methoxamine by >70% (*Figure 7A* and *B*; Supplementary material online, *Figure S9*). However, in arteries transfected with translation-blocking morpholinos targeted against NHE1, empagliflozin or dapagliflozin had a negligible effect on methoxamine-induced contractions (*Figure 7A* and *B*). Immunohistochemical studies on the same arteries revealed that targeting morpholinos reduced NHE1 staining in the adventitia considerably compared to scrambled morpholinos (*Figure 7C*). These studies corroborate our hypothesis that SGLT2 inhibitors relax mesenteric arteries through NHE1 inhibition-induced release of CGRP.

## 4. Discussion

This study investigated the underlying mechanism of SGLT2 inhibitor-induced vasorelaxation and identified a role for CGRP release from sensory nerves secondary to NHE1 inhibition. We show that structurally different SGLT inhibitors relaxed mesenteric arteries equipotently, divergent from their ability to block SGLT2 (or SGLT1)-mediated glucose transport.^1,23^ Relaxations produced by SGLT inhibitors were sensitive to TRPV1 and Kv7 channel blockade; however, electrophysiology recordings showed that SGLT2 inhibitors did not activate vascular Kv7 or TRPV1 channels directly. In addition, relaxations to dapagliflozin, empagliflozin, and the SGLT1 inhibitor mizagliflozin were attenuated by CGRP receptor blockade or by depletion of sensory nerve transmitters through capsaicin challenge. In contrast, SGLT2 inhibitors were poor relaxants of pre-contracted conduit renal and ineffective in septal resistance arteries where sensory nerves, evinced by staining for CGRP and TRPV1 in the adventitia, were absent compared to mesenteric arteries. The NHE1 blocker cariporide also relaxed mesenteric arteries, which was prevented by CGRP receptor and TRPV1 blockade, but had no effect in renal or septal arteries. Strikingly, NHE1 but not SGLT2 proteins co-localized with TRPV1 in the sensory nerves, and pre-application of cariporide or transfection with NHE1-targeted morpholino attenuated the inhibitory effects of empagliflozin and dapagliflozin.

The study provides strong evidence that SGLT2 inhibitors influence arterial reactivity by promoting the release of CGRP from sensory nerves. As the density of sensory nerve innervation varies across the vasculature and within an artery (see staining in *Figure 1*), this seminal finding explains much of the variability in data seen in previous publications.^9–11^ Interestingly, the effectiveness of zinc pyrithione, which also relaxes arteries through a release of CGRP from sensory nerves, was far greater in mesenteric arteries compared to renal and coronary arteries^35^ consistent with our observations with SGLT inhibitors. While SGLT2 is present in arterial smooth muscles, our pharmacological studies and comparative imaging suggest that the effects of the gliflozins are not mediated by an action on SGLT2 *per se* but via an additional effect of these agents on NHE1, as reported previously.^29–31^ This hypothesis was corroborated here by molecular knockdown of NHE1. Interestingly, a previous study in human visceral adipose arteries speculated NHE1 may be involved in the moderate relaxation produced by canaglifozin.^31^

### 4.1 SGLT2 inhibitors induce arterial relaxations

The present study shows that SGLT2 inhibitors relaxed pre-contracted mesenteric arteries at concentrations between 10 and 100 µM, in general agreement with previous work in mesenteric arteries and aortic rings.^7–11^ This is slightly higher than the therapeutic plasma concentrations in humans, which for empagliflozin is about 0.3 µM for the commonly prescribed dose of 10 mg and 8 µM for the maximum dose of 800 mg.^36^ But in an *ex vivo* setting, small changes in tension are hard to relate to the physiological impact of resistance and flow. It is common to use higher concentrations of drugs in *ex vivo* studies to delineate cellular processes. The present study shows that the mesenteric artery relaxations produced by dapagliflozin and empagliflozin, as well as mizagliflozin, were not sensitive to a range of K channel blockers (HMR-1556, iberiotoxin, 4-AP, TEA, and glibenclamide) but were sensitive to the pan-Kv7 channel blocker linopirdine, in agreement with earlier work in mesenteric arteries.^9–11^ However, our electrophysiological recordings showed that neither dapagliflozin nor empagliflozin directly enhanced potassium currents in oocytes expressing Kv7.4, Kv7.5, and KCNE4 (the combination in arterial smooth muscle^24^), suggesting that Kv7 channel activation is secondary to the release of a chemical intermediate. We have previously found that Kv7 channel-specific blockers impaired CGRP-induced relaxations in cerebral and mesenteric arteries^19–21^ so we propose that these channels are a functional endpoint of a relaxant cascade that involves CGRP release from sensory nerves.

### 4.2 The role of CGRP in arterial effects of SGLT2 inhibitors

Our hypothesis is that the arterial relaxation produced by the structurally dissimilar SGLT2 and SGLT1 inhibitors was mediated predominantly by CGRP release from perivascular sensory nerves.^14^ Thus, empagliflozin, dapagliflozin, or mizagliflozin relaxed mesenteric arteries that were impaired by blocking the CGRP receptor with BIBN-4096, although the lack of complete inhibition suggests other neuropeptides such as substance P may also be released. Immunohistochemistry with validated antibodies for TRPV1 or CGRP showed that mesenteric arteries had dense sensory nerve networks in the adventitia. In contrast, SGLT2 inhibitors were poor or ineffective relaxants in renal and septal arteries, respectively, that correlated with negligible or no TRPV1 or CGRP staining in the adventitia. The SGLT2 inhibitor-induced relaxations of mesenteric arteries were equally prevented by the application of the TRPV1 blocker AMG-517 or by depletion of CGRP through capsaicin treatment but not by the TRPA1 inhibitor AM0902. This suggests that the recruitment of TRPV1 rather than TRPA1 channels in sensory nerves is a key step in SGLT2 inhibitor-induced relaxations in the mesenteric artery.

TRPV1 is a polymodal cation channel regulated by various exogenous and endogenous activators. These include noxious chemicals (capsaicin or vanilloids), low pH (<6.0), high temperatures >43°C,^37^ lipid mediators (i.e. anandamide), lipoxygenase products (e.g. LTB4),^38^ and several signalling molecules (NGF, ATP, and PAR-2 agonists).^39,40^ The subsequent influx of cations through TRPV1 is sufficient to promote fusion of synaptic vesicles containing CGRP and other neuropeptides. Our data are consistent with SGLT2 inhibitors relaxing mesenteric arteries by promoting CGRP release in a manner dependent upon TRPV1 channels. However, in over-expression systems, dapagliflozin and empagliflozin failed to either activate TRPV1 currents or enhance the effect of low pH or capsaicin, suggesting that these agents do not work directly on the channel. Therefore, TRPV1 activation is a consequence of an additional mechanism.

### 4.3 NHE1 and arterial relaxation

NHE1 plays a primary role in cardiomyocytes and VSMCs to maintain cellular pH levels at ∼7.2.^41,42^ Altered NHE expression and activity have been linked to severe cardiac events, where during ischaemia, the pH change activates NHE, leading to cardiac injury.^42^ Many of the cardioprotective effects of SGLT2 inhibitors have been linked to reduced Na^+^ load and pH development in cardiomyocytes through an effect on NHE1,^29–31^ although this has been disputed.^43^ However, an arterial role for NHE1 suppression in the clinical benefit of SGLT2 inhibitors has not been demonstrated, although a role for NHE1 inhibition in relaxations of human visceral arteries by canagliflozin was speculated.^31^ NHE1 activation is linked to vasoconstriction and enhances the myogenic response in mouse resistance arteries.^44^ Increased NHE1 activity is also implicated in pulmonary artery hypertension, proliferation, and remodelling,^45^ with the protein regulating pH or acting as a protein anchor. We propose that NHE1 located in the sensory nerves also has a profound effect in arteries because they influence TRPV1 activity and the subsequent cation influx precipitates vesicular release of potent vasodilators including CGRP. Thus, in the present study, relaxations to SGLT2 inhibitors and the NHE1 blocker cariporide^30^ were prevented by a CGRP receptor antagonist and TRPV1 blocker. Moreover, pre-application of cariporide and, crucially, morpholino-based knockdown of NHE1 abrogated the response to dapagliflozin and empagliflozin. Interestingly, only NHE1 was identified in sensory nerves, unlike in the proximal convoluted tubule, where SGLT2 and NHE1 co-habit in the same microdomain.^46^ Inhibition of NHE1 in the smooth muscle cells would also influence the contractile state to some degree (see Boedtkjer *et al.*^26^), but our work provides robust evidence that SGLT2 inhibitors relax mesenteric arteries via TRPV1-dependent release of CGRP following NHE1 inhibition. Future studies will ascertain the precise mechanisms linking NHE1 inhibition and CGRP release.

**Translational perspective**

SGLT2 inhibitors like empagliflozin are effective hypoglycaemic agents but are also recommended treatments for heart failure and chronic kidney disease independent of circulating glucose. However, the mechanisms underlying the cardiovascular benefit are ill defined. The present study reveals that SGLT2 inhibitors relax arteries *ex vivo* via the release of calcitonin gene related peptide (CGRP) from sensory nerves, which is mediated by inhibition of sodium/hydrogen exchangers. The ensuing vasodilatation allied to anti-inflammatory, anti-fibrotic, and pro-inotropic actions of CGRP will ameliorate cardiovascular stress. This understanding of the mechanisms that underlie the arterial vasodilatation by these agents may inform future use.

## 5. Conclusion

CGRP is a potent vasodilator of many vascular beds,^14^ is a safeguard against cardiac ischaemia, and promotes cardiac contractility in failing hearts.^18^ Our data reveal that the beneficial effects of SGLT2 inhibitors likely stem from an ability to release cardioprotective CGRP into stressed circulations. The ensuing vasodilatation allied to anti-inflammatory, anti-fibrotic, and pro-inotropic actions of CGRP will support effective circulation and help to ameliorate cardiovascular stress.

## Supplementary Material

cvae156_Supplementary_Data

## Data Availability

The data underlying this article will be shared on reasonable request to the corresponding author.
