# Supplementary material for: Crucial role for sensory nerves and Na/H exchanger inhibition in dapagliflozin- and empagliflozin-induced arterial relaxation
Source: Cardiovasc Res. 2024 Jul 26;120(14):1811–24. doi: 10.1093/cvr/cvae156 (PMC11587556; doi:10.1093/cvr/cvae156)
Supplement: cvae156_Supplementary_Data [file cvae156_supplementary_data.docx]

Crucial role for sensory nerves and Na/H exchanger inhibition in dapagliflozin and empagliflozin-induced arterial relaxation.

Supplementary information

# **Methods**

## Animals

Experiments were performed on mesenteric, renal and coronary septal arteries from male rats aged 11-14 weeks and weighing 175-300g, housed within the Institute of Cancer Research facility (Surrey, UK). Rats were housed in cages with LSB Aspen woodchip bedding and a 12-hour light/dark cycle, maintained at constant temperature (21±1°C) and humidity (50%±10%), with free access to water and food, all in accordance with the Animal Scientific Procedures Act 1986 (ASPA). The tissues were transported each day to St George’s University of London, bathed and preserved in a salt solution containing (mmol·L^-1^): 126 NaCl, 6 KCl, 10 Glucose, 11 HEPES, 1.2 MgCl_2_ and set to pH7.4 with NaOH. Animals were sacrificed by cervical dislocation and death confirmed via severing the femoral artery circulation in accordance with Schedule 1 of the ASPA 1986.

Second order mesenteric and the left and right main renal arteries were dissected, cleaned of fat and adjacent tissue and stored on ice in physiological Salt solution (PSS – see 1.9 solutions).

## Myography

All arteries were cut into ~2mm segments and mounted on 40µm stainless steel wires in a myograph (DMT, Aarhus, Denmark). The myograph chambers contained PSS that was bubbled with 95% oxygen and 5% carbon dioxide at 37°C. Tension in each segment was recorded using LabChart Pro Software (ADInstruments, Oxford, UK).

Arteries were cut into ~2mm segments and mounted on 40µm stainless steel wires in a myograph (DMT, Aarhus, Denmark). The myograph chambers contained physiological salt solution (PSS, composition in Supplement section 1.9) that was bubbled with 95% oxygen and 5% carbon dioxide at 37°C. Tension in each segment was recorded using LabChart Pro Software (ADInstruments, Oxford, UK). All vessels were subject to a normalisation procedure^22^ to standardise the experimental conditions and arteries were set to an internal circumference 90% of the diameter at in-vivo transmural pressure (13.3 kPa or 10.3 kPa for septal arteries). Endothelial integrity was estimated by the response to 10µM carbachol applied to arteries constricted with 10µM of the α1-adrenoreceptor agonist, methoxamine. The endothelium was denuded by mechanical abrasion with an eyebrow hair and effectiveness of removal ascertained by a carbachol challenge in all experiments with septal arteries.

Arterial segments were preconstricted with 10 µM methoxamine and responses to the SGLT2 inhibitors dapagliflozin, empagliflozin, ertugliflozin (1 to 100 µM), the SGLT1 inhibitor mizagliflozin (1-30µM), NHE1 inhibitor cariporide, CGRP (10pM- 10nM) and capsaicin (10 µM) determined. Vessels were pre-incubated in the presence or absence of a combination of solvent control dimethyl sulphoxide (DMSO) or pharmacological agents: Linopirdine (pan Kv7 channel blocker, 10µM), BIBN-4096 (CGRP receptor blocker, 1µM), Capsaicin (TRPV1 agonist, 10µM), AMG-517 (TRPV1 blocker, 1µM), AM0902 (TRPA1 channel blocker, 1 and 10 µM), HMR-1556 (Kv7.1 channel blocker, 10µM), Iberiotoxin (BK_Ca_ channel blocker, 100nM), 4-aminopirydine (4-AP; 1mM), tetraethylammonium (TEA; 1mM) and glibenclamide (K_ATP_ channel blocker, 1 µM and 3 µM). To deplete sensory nerves relaxed arteries were challenged with 10µM capsaicin for 5 mins followed by extensive washout over 10 mins. This was repeated twice before contraction with methoxamine. A similar protocol was used for the cariporide pretreatment experiments, except cariporide was left in the bath at the time of contraction.

## Immunohistochemistry

After completion of functional myography experiments, mesenteric and renal arterial segments were fixed *in situ* in myograph chambers with 4% paraformaldehyde (J61899, Thermo Scientific) for 1 hour at room temperature. Fixed arteries were washed with PBS, removed from wire myograph chambers by cutting open laterally and then separated into 2 fixed segments. Arteries were blocked for 90 minutes at room temperature with blocking buffer (1% BSA, 0.5% Triton X-100, 0.05% Tween 20 in PBS) and incubated overnight at 4°C with guinea pig anti-TRPV1 (Ab10295, 1:1000, Abcam) and goat anti-CGRP (Ab36001, 1:1000, Abcam), rabbit anti-NHE1 (PA5115917, 1:1000, Invitrogen), mouse anti-SGLT2 (sc-393350, 1:200, Santa Cruz), rabbit anti-smooth muscle Myosin heavy chain 11 (ab125884, 1:500, Abcam) and Goat anti mouse CD31/PECAM-1 (AF3628-SP, 1:150, R&D Systems) diluted in blocking buffer. This was followed by a secondary antibody incubation with either goat anti-guinea pig (Alexa fluor 488, A11073, Life Technologies), donkey anti-goat (Alexa fluor 633, A21082, Life Technologies), donkey anti-rabbit (Alexa fluor 488, A21206, Life Technologies) or donkey anti-mouse (Alexa fluor 594, A21203, Invitrogen) diluted in blocking buffer for 90 minutes at room temperature. Arteries were then placed in mounting medium (Vectasheild plus Antifade, Vector Laboratories) and laid flat between 2 glass coverslips. Arteries were excited at 405, 488, 536 and 635 nm and fluorescence acquired through a water immersion objective (1.15, NAI, 1024 x 1024 pixels, x40 lens objective, Olympus) using a Fv1000 laser scanning confocal microscope (Olympus, Southend-on-Sea, UK). Z stacks were taken through every arterial segment starting from the arterial wall in 1µM increments using Fluaview version 4.1 and Imaris version 8.0.2 Bitplane software.

## Immunocytochemistry (ICC)

Vascular smooth muscle cells were isolated from whole mesenteric and renal arteries from Wister rats. Arteries were placed in a smooth muscle dissociation solution (SMDS- see solutions for composition) supplemented with 1.75 mg·ml−1 Collagenase Type IA, 0.9 mg·ml^−1^ protease, 1 mg·ml^−1^ trypsin inhibitor and 1 mg·ml^−1^ bovine serum albumin (Sigma, UK) at 37°C for 45 minutes. Vessels were washed in SMDS and underwent mechanical trituration using a glass Pasteur pipette to liberate vascular smooth muscle cells. The subsequent cell suspension was plated onto 13mm coverslips and left at room temperature (RT, 20-22°C) for 45 minutes to adhere. The VSMCs were fixed with 4% paraformaldehyde for 15 minutes and then stored in a 24-well plate with filtered PBS at 4°C until used for ICC experiments.

The VSMCs were incubated in WGA Texas Red membrane stain (Thermos Fisher Scientific, Invitrogen) for 10 minutes. Cells were washed with PBS and incubated in 100 mmol·L−1 glycine in PBS for 5 minutes and subsequently in blocking solution (PBS containing 0.1% Triton X-100 and 10% FBS in PBS) for 45 minutes. Cells then underwent an overnight incubation with a mouse anti-SGLT2 antibody (dilution 1:200, Santa Cruz, Texas, USA), then washed in PBS and incubated with a donkey anti-mouse secondary antibody conjugated to Alexa Fluor 488 (dilution 1:100, Thermo-Fisher, Paisley, UK). The coverslips were washed in PBS before being mounted onto a microscope slide with 4’,6-diamidino-2-phenylindole (DAPI) (Vectashield, Vector Laboratories, Burlingame, CA) mounting medium.

## Western Blot

Whole protein lysates from mesenteric and renal arteries were prepared using Triton Buffer (Fisher Scientific) supplemented with protease and phosphatase inhibitors (cOmplete, mini and PhosSTOP from Roche). Protein concentrations were determined via the Pierce™ BCA Protein Assay Kit (Thermo Fisher Scientific, Loughborough, UK). The samples were run under reducing conditions with 4-12% Bolt™ Bis-Tris Plus pre-cast gels (Invitrogen) and proteins were transferred to a nitrocellulose membrane. Membranes were blocked for at least 0.5 hour in 3% BSA-PBS and incubated overnight at 4°C with the primary SGLT2 mouse monoclonal antibody (D-6, sc-393350, 1/200 dilution, Santa Cruz). The membranes were incubated with highly adsorbed horseradish peroxidase-conjugated goat anti-mouse IgG (A16078, Fisher Scientific) for 1h at RT and developed using Immobilon™ Western Chemiluminescent HRP Substrate (Millipore). Detection and quantification of chemiluminescence intensities were quantified by using Chemidoc^TM^ imaging system and Image Lab 5.2.1 software (BioRad). Blots were stripped with restore buffer (Fisher Scientific) according to the manufacturer’s instructions and re-probed with a rabbit-anti-actin antibody (#4970, Cell Signalling Technology) and highly-adsorbed horseradish peroxidase-conjugated donkey-anti rabbit IgG (Merks) as above.

## Cell Cultures

HEK293 cells (ATCC® CRL-1573) were cultured in a complete growth medium containing Dulbecco's modified Eagle's medium with high-Glucose (DMEM GibcoTM) complemented with 10% fetal bovine serum (HyCloneTM) and 100 U/ml of Penicillin-Streptomycin (GibcoTM). Cell cultures were maintained in a humidified incubator at 37°C with an atmosphere of 95% of air and 5% of CO_2_. Cells were subcultured every 3 days using 0.25% (w/V) Trypsin-EDTA solution (GibcoTM).

## Electrophysiology

The effect of various SGLT2 inhibitors was assessed on currents generated by the over-expression of Kv7 and TRPV1 genes in *Xenopus laevis* oocytes and HEK293 cells respectively.

### Channel subunit cRNA preparation and *Xenopus laevis* oocyte injection

We generated cRNA transcripts encoding human Kv7.4, Kv7.5 and KCNE4 by in vitro transcription using the mMessage mMachine kit (Thermo Fisher Scientific, Waltham, MA, USA) according to manufacturer’s instructions, after vector linearization, from cDNA sub-cloned into expression vectors (pTLNx and pXOOM) incorporating *Xenopus laevis* β-globin 5’ and 3’ UTRs flanking the coding region to enhance translation and cRNA stability. We injected defolliculated stage V and VI *Xenopus laevis* oocytes (Xenoocyte, Dexter, MI, USA) with Kv7 and KCNE4 cRNAs (2-10 ng) and incubated the oocytes at 16 ^o^C in ND96 oocyte storage solution containing penicillin and streptomycin, with daily washing, for 2-5 days prior to two-electrode voltage-clamp (TEVC) recording.

## Two-electrode voltage clamp (TEVC)

We performed TEVC at room temperature using an OC-725C amplifier (Warner Instruments, Hamden, CT, USA) and pClamp10 software (Molecular Devices, Sunnyvale, CA, USA) 2-5 days after cRNA injection. We placed oocytes in a small-volume oocyte bath (Warner) and viewed them with a dissection microscope for cellular electrophysiology. We sourced chemicals from Sigma-Aldrich (St. Louis, MO, USA). SGLT2 inhibitors were introduced into the oocyte recording bath by gravity perfusion at a constant flow of 1 ml per minute for 3 minutes prior to recording. Pipettes were of 1-2 MΩ resistance when filled with 3 M KCl. Currents were recorded in response to voltage pulses between -80 mV and +40 mV at 20 mV intervals from a holding potential of -80 mV, to yield current-voltage relationships. Data were analyzed using Clampfit (Molecular Devices) and Graphpad Prism software (GraphPad, San Diego, CA, USA), stating values as mean ± SEM. Raw tail currents were plotted versus prepulse voltage and fitted with a single Boltzmann function:

Eq. 1:

$$\boldsymbol{g=}\frac{\mathbf{(}\boldsymbol{A}_{\boldsymbol{1}}\boldsymbol{-}\boldsymbol{A}_{\boldsymbol{2}}\boldsymbol{)}}{\left\{ \boldsymbol{1 + exp [}\boldsymbol{V}_{\frac{\boldsymbol{1}}{\boldsymbol{2}}}\boldsymbol{-V/Vs]} \right\}\boldsymbol{y+}\boldsymbol{A}_{\boldsymbol{2}}}$$

where g is the normalized tail conductance, A_1_ is the initial value at -∞, A_2_ is the final value at +∞, V_1/2_ is the half-maximal voltage of activation and Vs the slope factor.

### Transient cell transfection and patch-clamp

For patch-clamp experiments, the cells were seeded on poly-d-lysine (1 mg/ml, Sigma) coated coverslips for patch-clamp experiments. After 24 hrs, HEK293 cells were transfected with plasmids pcDNA3.1 containing the rat wild-type TRPV1 channel (1.5 µg) and pcDNA3.1 with green fluorescent protein (GFP, 500 ng) to identify successfully transfected cells using jetPEI™ Polyplus transfection reagent, per manufacturer's instructions.

TRPV1 currents were recorded from transiently transfected HEK-293 using the patch-clamp technique in the outside-out configuration (Hamill et al., 1981). Solutions were changed with an RSC-200 rapid solution changer (Molecular Kinetics). Currents were low-pass filtered at 2 kHz and sampled at 10 kHz with an EPC 10 amplifier (HEKA Elektronik) and were plotted and analysed with Igor Pro (Wavemetrics Inc.). The capsaicin stock was prepared at 10 mM in absolute ethanol and then diluted to 250 nM in recording solution. Experiments were performed at room temperature (24 °C) and mean current values were measured after channel activation had reached the steady-state. Pipettes were fabricated with borosilicate glass and had a resistance of 5 MΩ.

Currents were obtained using voltage protocols where the holding potential was 0 mV and then stepped from -120 to 120 mV for 100 ms using a square voltage protocol. Briefly, leak currents were obtained in the absence of agonists or dapagliflozin and then excised membrane patches containing TRPV1 channels were exposed to DAPA, capsaicin, capsaicin + DAPA, pH 6 or pH 6 + DAPA. All currents were leak-subtracted and normalized to currents activated either by capsaicin or pH alone.

### Solutions

Physiological salt solution (PSS) contained (mM): 119 NaCl, 4.5 KCl, 1.17 MgSO_4_·7H_2_0, 1.18 NaH_2_PO_4_, 25 NaHCO_3_, 5 glucose, 1.25 CaCl_2_.

Smooth muscle dissociation solution (SMDS) contained (mM); 120 NaCl, 6 KCl, 12 glucose, 10 HEPES and 1.2 MgCl_2._

PBS contained (mM) 10 mM phosphate buffer, 2.7 mM KCl, 137 mM NaCl, pH 7.4.

Intracellular and extracellular recording solutions for TRPV1 recordings in HEK cells contained (in mM): 130 NaCl, 3 HEPES (pH 7.2) and 1 EDTA for experiments at pH 7.2 and HEPES was replaced by 3 mM MES for experiments were TRPV1 was activated by pH 6.

External solution for Kv7 channel recording in xenopus oocytes contained (in mM): 96 NaCl, 4 KCl, 1 MgCl2, 1 CaCl_2_, 10 HEPES (pH 7.6).


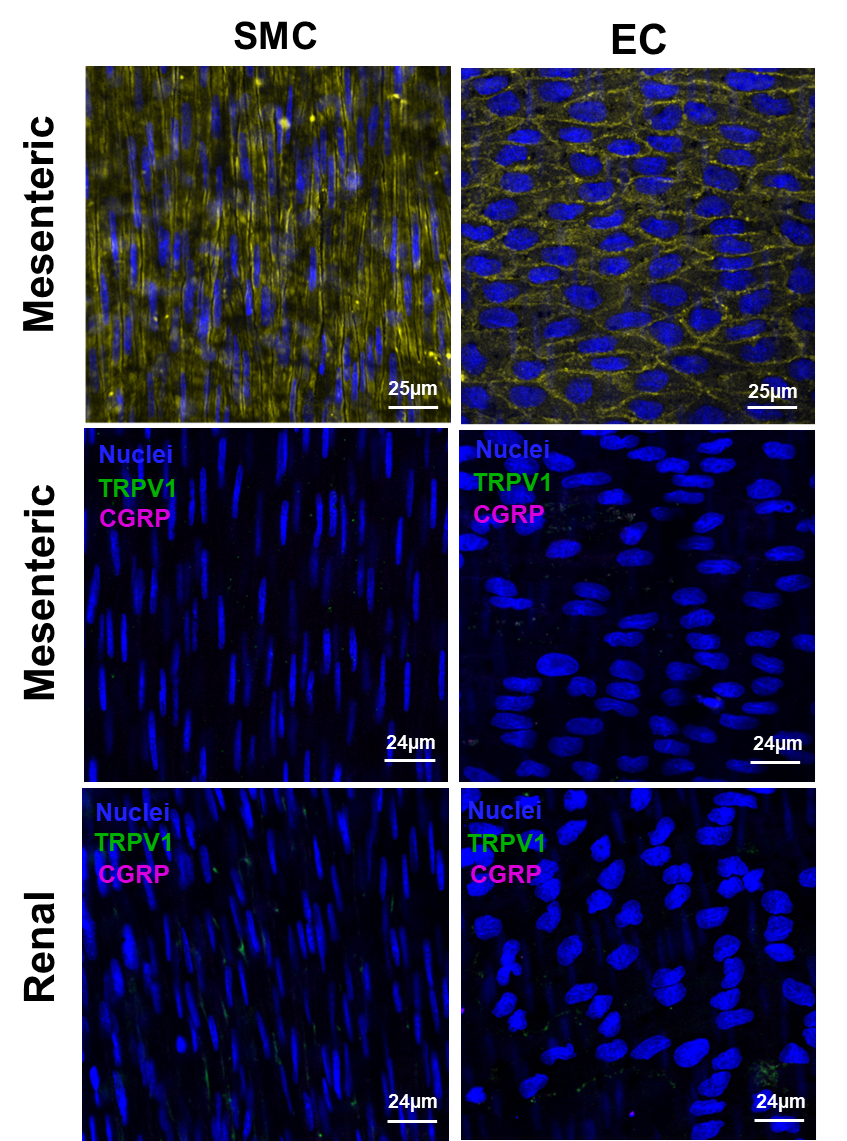


**Supplement Figure 1. Lack of TRPV1 and CGRP expression in mesenteric and renal smooth muscle cells (SMC) and endothelial cell (EC) layers.**

The top panel in yellow shows representative staining for the myosin heavy chain (smooth muscle marker) and CD31 (endothelial cell marker). The middle row shows no TRPV1 (green) or CGRP (magenta) in the smooth muscle cells (SMC) and endothelial cells (EC) of mesenteric arteries and the bottom row shows renal arteries. Nuclei were labelled in blue (representative of N=4).


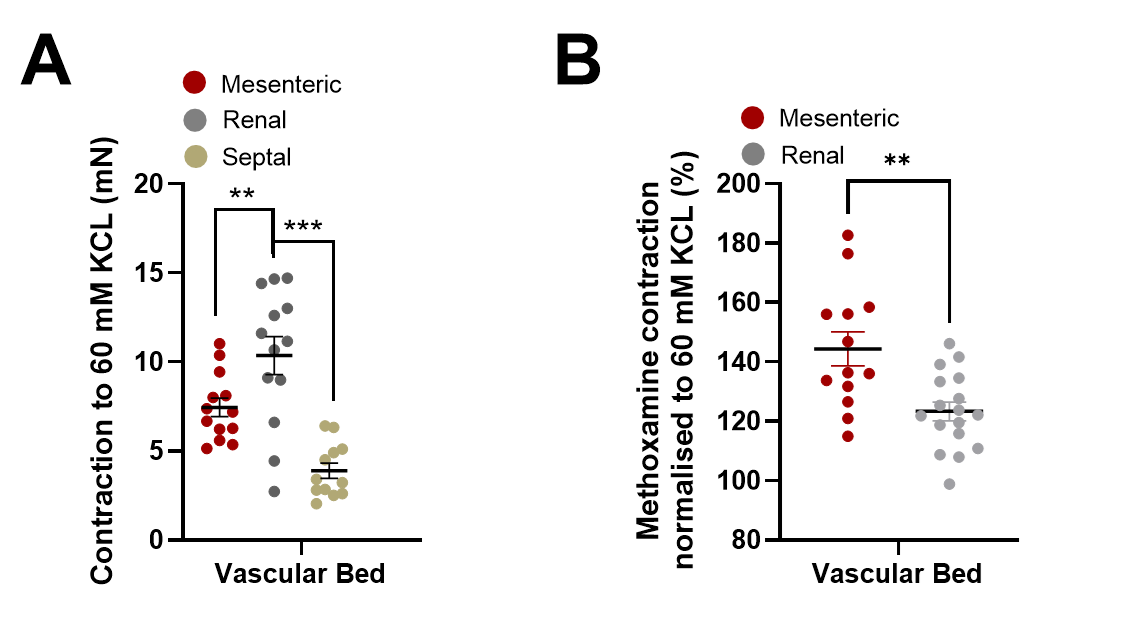


**Supplement figure 2. The contraction to KCL and methoxamine in all 3 artery beds.**

The contraction to 60 mM KCL in raw mN from mesenteric (red, N=14), renal (grey, N=13) and septal (beige, N=12) arteries is shown in panel A. Panel B shows the contraction to 10µM methoxamine normalised as a percentage of the contraction to KCl in mesenteric (red, N=13), renal (grey, N=17) arteries. Data is shown as Mean ±SEM denoted by error bars, and a non-parametric Mann-Whitney t-test was used to generate significant values (**=P<0.01, ***=P<0.001) (N= number of animals used).

**Supplement Figure 3. Lack of correlation between SGLT2 inhibitor and carbachol response in mesenteric arteries**

The percentage relaxation to 30 µM dapagliflozin (A, N=33) and empagliflozin (B, N=16) in mesenteric arteries with positive endothelium (>90%) and denuded (<10%). All values are expressed as mean ± SEM denoted by the error bars, and a non-parametric Mann-Whitney t-test was used to generate significant values.


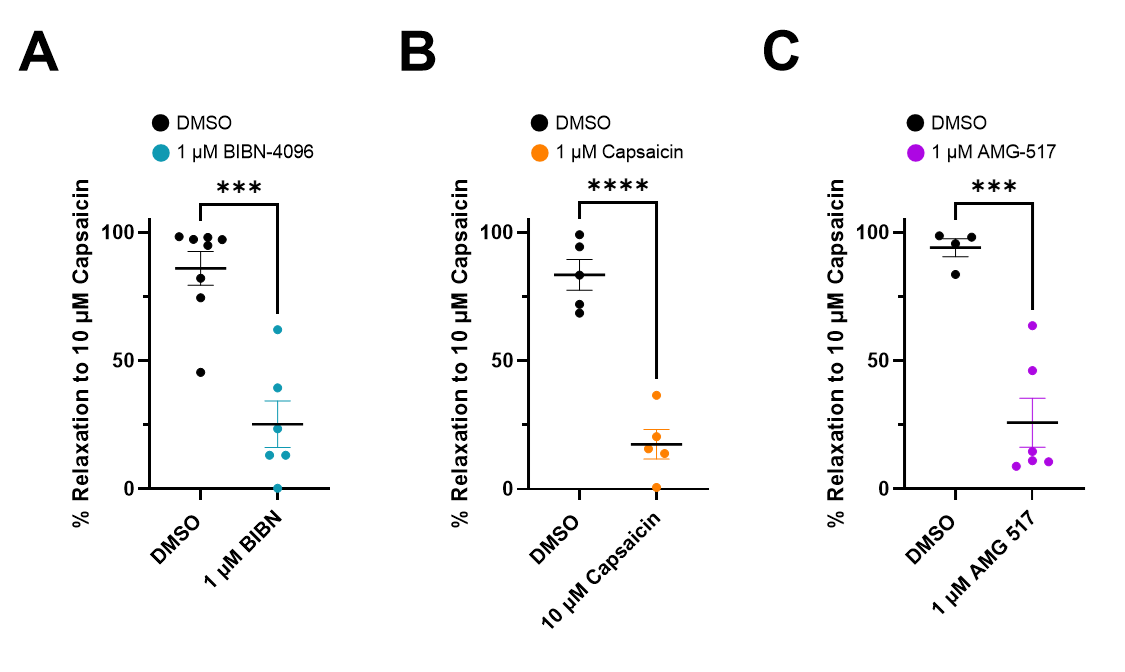


**Supplement figure 4. Effect of CGRP receptor blocker, TRPV1 blocker and capsaicin incubation on capsaicin-induced relaxations.**

The mean effect of 10 µM capsaicin in arteries in the presence and absence of 1 µM CGRP blocker BIBN (A, N=6-8) or after sensory nerve depletion (B, 3-5 min pulses of capsaicin then wash out, N=5) or in the presence of the TRPV1 blocker AMG-517 (C, N=5). All values are expressed as mean ± SEM denoted by the error bars. A two-way statistical ANOVA with a post-hoc Sidak test was used to generate significant values (*=P<0.05, **=P<0.01, ***=P<0.001, ****=P<0.0001) (N= number of animals used).

**Supplement figure 5. Effect of TRPA1 blocker and KATP blocker on dapagliflozin and empagliflozin induced relaxations.**

The mean response (1µM-100µM) to dapagliflozin (A, N=5) and empagliflozin (B, N=5) when pre-incubated with either DMSO (black), 10 µM AM0902 (Green) or 3 µM Glibenclamide (orange) (N=5). All values are expressed as mean ± SEM, where N=(number of animals used).

**Supplement figure 6. Mean amplitude of methoxamine evoked contractions.**

(A) The mean amplitude of the contraction produced by 10µM Methoxamine under the different conditions pre-incubated with 10µM Capsaicin (orange) or 1µM AMG-517 (Purple, N= 5-10). (B) The mean amplitude of the contraction produced by 10µM Methoxamine in the presence and absence of 10µM Cariporide (Green, N=5-6). Data is shown as mean contraction (mN) ± SEM, where N= (number of animals).


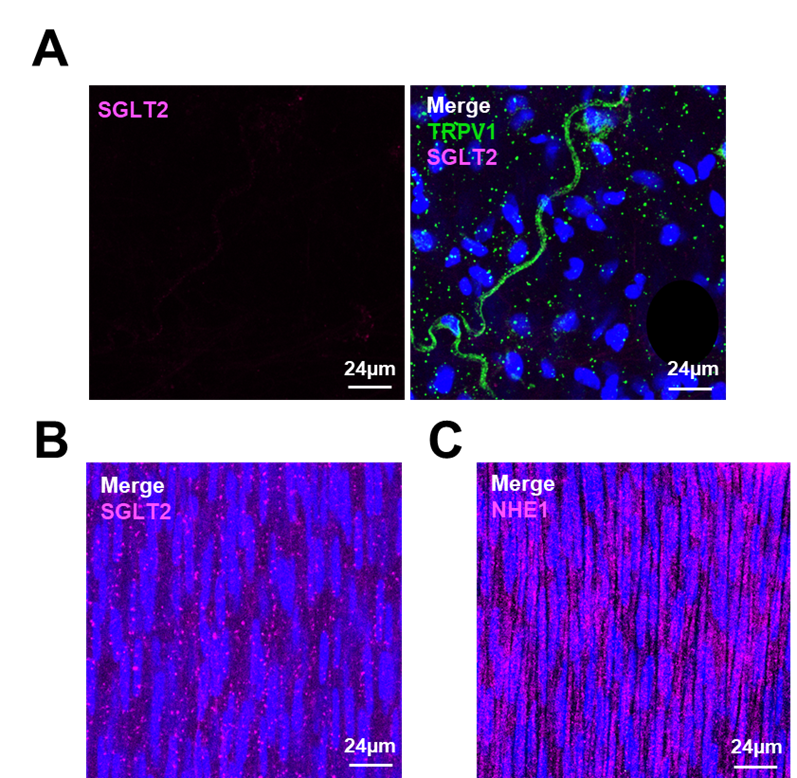


**Supplement figure 7: Expression of NHE and SGLT2 in mesenteric artery layers​.**

Immunohistochemistry labelling of SGLT2 (magenta) with TRPV1 (Green) in panel A in the adventitia of whole mesenteric arteries. Labelling of SGLT2 (magenta) in panel B and NHE1 in panel C (magenta) in smooth muscle cell layer of mesenteric arteries. Nuclei were labelled in blue. Images are representative of 3 such experiments.​


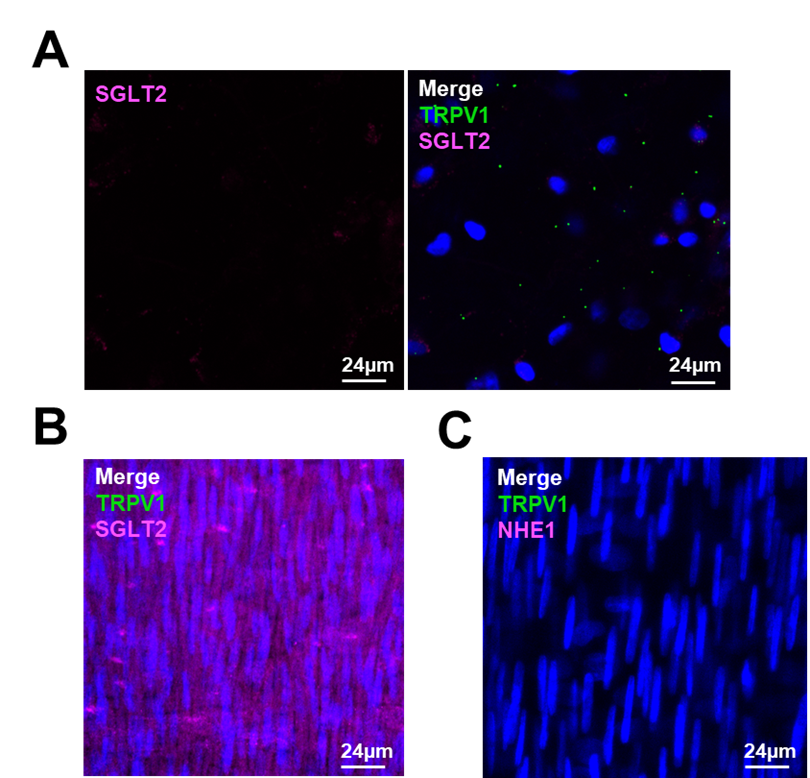


**Supplement figure 8: Lack of expression of NHE and SGLT2 in renal artery adventitia.**

Immunohistochemistry labelling of SGLT2 (magenta) with TRPV1 (Green) in panel A in the adventitia of whole renal arteries. Labelling of SGLT2 (magenta) in panel B and NHE1 in panel C (magenta) in the smooth muscle cell layer of renal arteries. Nuclei were labelled in blue. Images are representative of 3 such experiments.​

**Supplement figure 9: Mean amplitude of methoxamine evoked contractions in morpholino incubated vessels compared to fresh vessels.**

Contraction to 10µM methoxamine in scrambled control morpholinos (black, N=10) compared to targeted morpholino (Green, N=10) and fresh arteries (grey, N=13). Data is shown as Mean ±SEM denoted by error bars, and a non-parametric Mann-Whitney t-test was used to generate significant values (**=P<0.01), (N= number of animals used).
